# Prevalence and obstetric risk factors of stress urinary incontinence in parous women: a cross-sectional study in rural areas of Mangaluru, India

**DOI:** 10.7717/peerj.21722

**Published:** 2026-09-08

**Authors:** Amisha S. Amin, Kunnath Chacko Leena

**Affiliations:** 1 Assistant Professor, Department of OBG Nursing, Yenepoya Nursing College, Yenepoya (Deemed to be University), Derlakatte, Mangalore, Karnataka, India; 2 Principal/Dean, Yenepoya Nursing College, Yenepoya (deemed to be University), Derlakatte, Mangalore, Karnataka, India

**Keywords:** Prevalence, Stress urinary incontinence, Obstetric risk factors, Parous women, Rural India

## Abstract

**Background:**

Stress urinary incontinence (SUI) is common among parous women, yet community-level data from rural India remain limited. This study estimated the prevalence of SUI among relatively healthy, rural, reproductive-age parous women without major comorbidities.

**Methods:**

A community-based cross-sectional study was conducted among 412 parous women (aged 25–40 years) using proportionate stratified random sampling across seven rural wards. SUI was assessed using the modified International Continence Society (ICS) Uniform Cough Stress Test (CST) with a standard bladder-fill protocol. Obstetric data were collected via a validated questionnaire. Bivariate and multivariable associations were analysed using modified Poisson regression with robust standard errors to estimate crude prevalence ratios (cPR) and adjusted prevalence ratios (aPR).

**Results:**

The prevalence of SUI was 53.4% (*n* = 220), yet 98.3% (*n* = 405) of participants had no prior information regarding its management. The relatively high prevalence may reflect the use of an objective modified ICS-based cough stress test, which can identify previously unreported cases. Maternal age was independently associated with higher SUI prevalence in the adjusted modified Poisson regression model (aPR = 1.198 per year; 95% CI [1.168–1.229]; *p* < 0.001).

**Conclusion:**

SUI affects more than half of relatively healthy, reproductive-age parous women in this rural setting; however, these findings represent associations within a clinically screened population and should not be interpreted as causal relationships or generalised to all rural women. Increasing maternal age was independently associated with higher SUI prevalence, supporting consideration of routine pelvic floor assessment and targeted community-based education within primary maternal healthcare services.

## Introduction

Stress urinary incontinence (SUI) refers to the unintentional leakage of urine during activities that increase intra-abdominal pressure, such as coughing, sneezing, or physical exertion (Anand, Khan & Khan, 2023; Pandey et al., 2019). As classified by the International Continence Society (ICS), it is a clinically significant subtype of urinary incontinence (Pandey et al., 2019). Global prevalence estimates vary widely, ranging from 20% to 60% across diverse populations (Moris et al., 2025; Charpot & Sagar, 2021; Li, Li & Wu, 2023; Rubilotta et al., 2019), affecting an estimated 200 million individuals worldwide (Hu & Pierre, 2019; Pradeep & Anand, 2023). While not life-threatening, SUI profoundly impacts quality of life, often causing physical discomfort, diminished emotional well-being, and social withdrawal (How et al., 2022; Tomar & Sharma, 2021).

In India, urinary incontinence represents a significant yet overlooked public health issue. Social taboos and limited healthcare access—particularly in rural areas—contribute to a high proportion of unreported cases (Kumar et al., 2022). In many patriarchal communities, open discussion of intimate health problems such as urinary leakage remains stigmatized. Consequently, many women normalize their symptoms, perceiving them as an inevitable part of aging or the aftermath of childbirth, thereby delaying appropriate care-seeking (Pradeep & Anand, 2023). Research from urban centres in India, largely based on self-reported symptoms, suggests a SUI prevalence among parous women of approximately 30%–40% (Sharma, Khandhedia & Dave, 2022; Soni & Bindra, 2023). However, there is a marked paucity of community-based data from rural populations, making it difficult to generalise these findings to the wider Indian female population. Unlike hospital-based studies, which may be subject to selection bias by capturing only symptomatic or treatment-seeking individuals, a community-based approach provides a more accurate reflection of the true burden of SUI in the general rural population. Approximately two-thirds of Indian women of reproductive age reside in rural areas, underscoring the importance of addressing women’s health issues in rural communities (International Institute for Population Sciences (IIPS) and ICF, 2021).

While SUI is multifactorial, a primary underlying cause is the weakening of the pelvic floor muscles and connective tissues (Falah-Hassani et al., 2021). Among the various contributing factors, pregnancy and vaginal delivery are considered the most significant. The combination of sustained intra-abdominal pressure during pregnancy and mechanical trauma during childbirth can compromise pelvic support structures, predisposing women to SUI (De Lancey & Pipitone, 2024). Advancing maternal age, multiple vaginal deliveries, prolonged second stage of labour, fetal macrosomia, and instrumental deliveries such as forceps or vacuum extraction have all been associated with increased risk of pelvic floor dysfunction (Yang & Liao, 2022; Syed & Hofer, 2023). Without adequate perineal support during childbirth or appropriate postnatal pelvic floor rehabilitation, these physiological stresses may result in lasting anatomical changes and the subsequent development of SUI (Frohlich & Kettle, 2015). Focusing on women aged 25–40 years allows for the assessment of SUI during peak reproductive years. By selecting a relatively healthy, younger cohort, we aimed to study SUI prevalence in a group where the confounding effects of the menopausal transition and major age-related systemic illnesses are naturally less prevalent.

Existing rural data, though limited, indicate prevalence rates of 30% to 45% (Sharma, Khandhedia & Dave, 2022; Soni & Bindra, 2023); however, these estimates often reflect older or postpartum populations and often rely on subjective screening tools. In contrast, this study employs the modified ICS Cough Stress Test (CST) to provide an objective clinical confirmation. This community-based approach is essential for establishing baseline data to develop targeted interventions, such as integrating pelvic health education into routine maternal care. Therefore, this study aimed to estimate the prevalence of SUI and identify its associated obstetric and biological risk factors among parous women aged 25–40 years in rural Mangaluru, India.

## Methods and Materials

A community-based cross-sectional study was conducted between September and December 2024 in Ullal Taluk, Mangaluru, Karnataka, India. This study represents Phase I (baseline epidemiological assessment) of a larger research programme designed to evaluate a pelvic floor muscle–focused bladder training intervention among parous women with stress urinary incontinence (SUI). The present phase aimed to estimate the prevalence of clinically confirmed SUI and to examine associations with selected obstetric characteristics within a defined community population.

Stress urinary incontinence is recognised as a multifactorial condition, influenced by obstetric, lifestyle, medical, and hormonal factors. Accordingly, this study did not attempt to treat obstetric factors as the sole determinants of SUI. Instead, restriction was employed as a design strategy to examine these associations within a clinically coherent subgroup, specifically, women who would be ideal candidates for a pelvic floor–based behavioural intervention. Consequently, findings are interpreted as associations within this defined population, rather than as direct causal effects.

**Figure 1:**
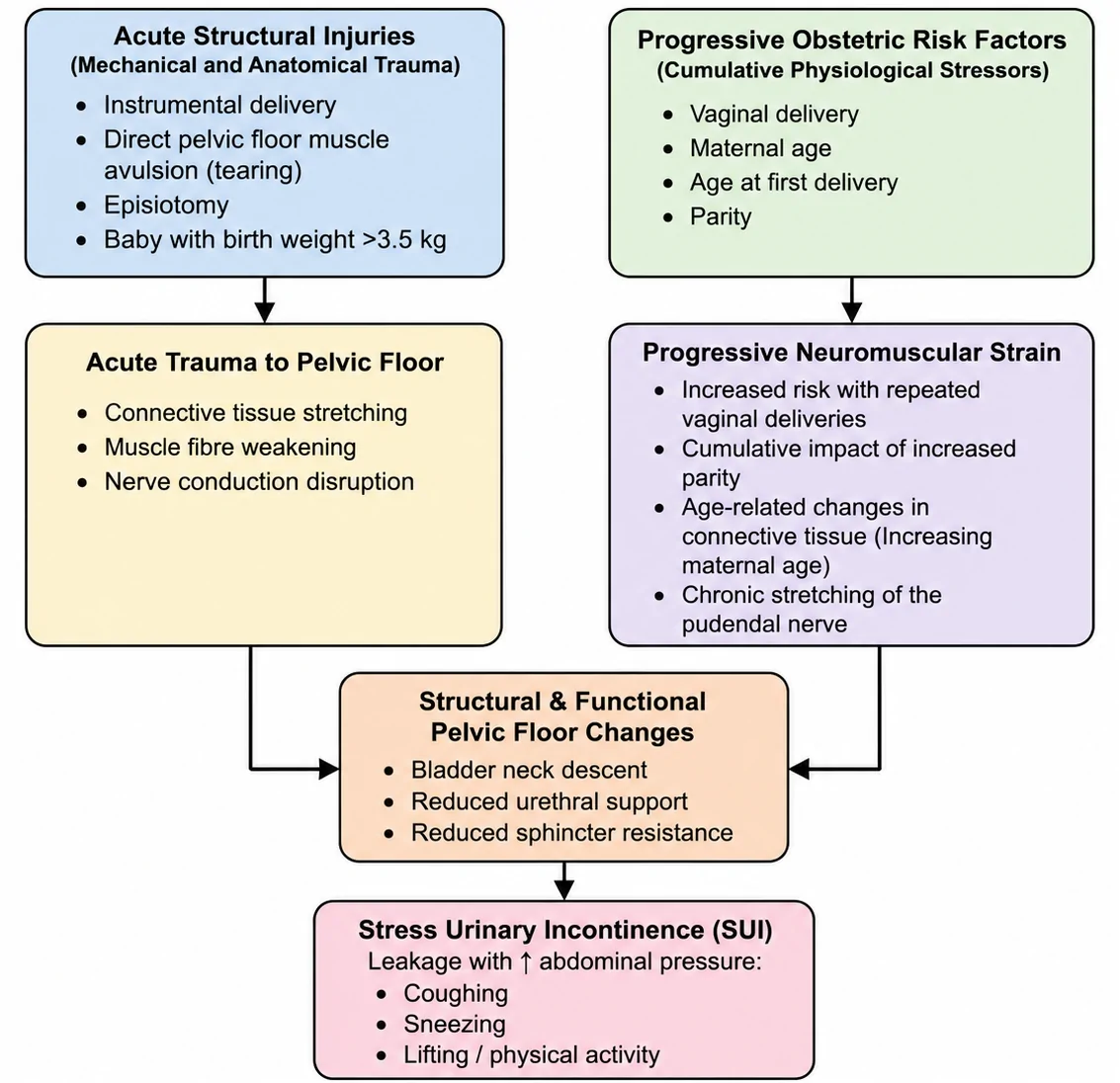
Conceptual framework of acute and progressive obstetric determinants of stress urinary incontinence.

The study was conducted in rural service areas of two Primary Health Centres (PHCs), where community-level data on SUI are limited and routine screening is uncommon. A conceptual framework was developed to depict the hypothesised relationships between key obstetric exposures—such as age at first delivery, parity, mode of delivery, episiotomy, perineal trauma, and baby’s birth weight—and the development of SUI (Fig. 1). The conceptual framework illustrates parallel pathways leading to stress urinary incontinence (SUI). The direct pathway involves acute structural birth injuries (*e.g.*, instrumental delivery, macrosomia, and perineal trauma), causing immediate anatomical disruption. The indirect pathway involves progressive factors, including chronological ageing and parity, which contribute to cumulative neuromuscular strain. Both pathways converge on structural and functional changes that result in the clinical manifestation of SUI during increased intra-abdominal pressure.

### Sample size and sampling

The sample size was primarily powered for prevalence estimation. The sample size was calculated using the formula for a single proportion based on an expected SUI prevalence of 55.5% (*p* = 0.555) from a recent regional study (Rajalakshmi et al., 2024). Assuming a 95% confidence level and 5% absolute precision, the minimum required sample was 380 participants. To account for the stratified sampling design, a design effect (DEFF) of 1.05 was applied, reflecting the expected similarity across rural wards. To ensure proportionate representation across wards and account for potential non-response, the final target sample was increased to 412. While the study was sufficiently powered for the primary objective of prevalence estimation, the analysis of specific obstetric risk factors was treated as a secondary objective and was subject to the available variability within this sample size.

### Sample selection

#### Study setting and participant selection

The study was conducted in two Primary Health Centres (PHCs) in Ullal Taluk. Seven rural wards within these service areas were selected, with each ward designated as a stratum. A total sampling frame of *N* = 736 eligible parous women, based on history of vaginal delivery and age, was identified from community health registries. To ensure community representation, the target of 412 was distributed across the seven wards using proportionate allocation.

**Figure 2:**
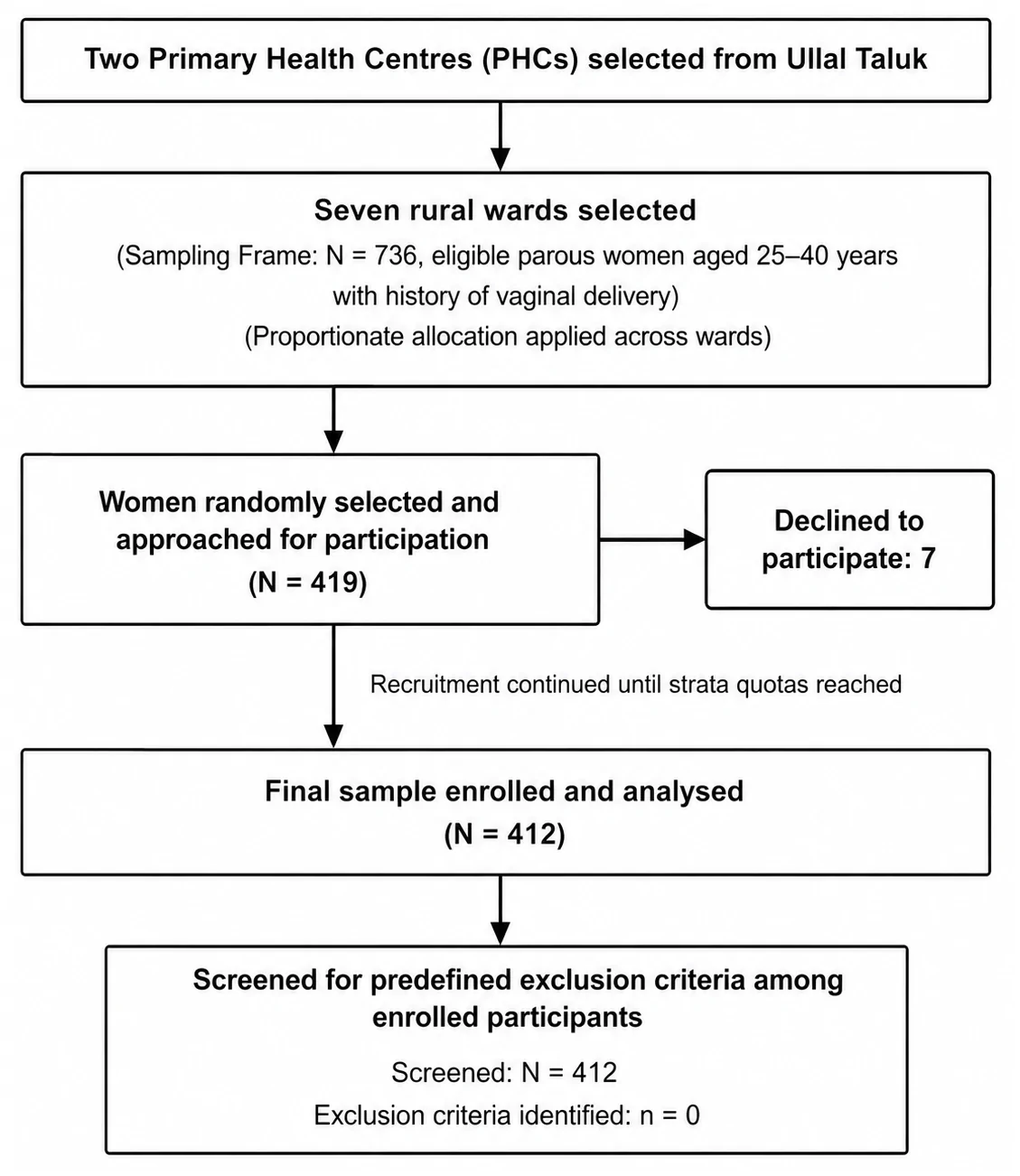
Participant flow diagram showing sampling frame, selection, exclusions, and final analytical sample.

Within each stratum, participants were selected using simple random sampling. To reach the required quota while maintaining proportionate balance, sequential recruitment was employed: when a selected individual declined (*n* = 7), the next randomly selected woman from the same ward’s registry was approached. Reasons for initial refusal were not formally recorded but typically related to time constraints rather than the nature of the study. This process continued until the pre-determined sample of 412 consenting participants was reached and analysed. Notably, once consent was obtained, there was a 100% completion rate for both the interview and the clinical cough stress test, with no dropouts or refusals of specific sensitive items reported during the assessment phase (Fig. 2).

To build rapport and minimise recruitment bias and dropout, a local community health worker assisted with initial introductions. To ensure data integrity and minimise social desirability bias, the clinical assessments were conducted independently by the Principal Investigator (a Registered Nurse). All 412 enrolled participants completed both the interview and the clinical assessment.

### Eligibility criteria

The study specifically aimed to establish the prevalence of SUI among otherwise healthy, non-obese reproductive-age women, isolating the “silent” burden of pelvic floor dysfunction directly attributable to obstetric trauma. This focus ensured that the findings were clinically relevant for preventive pelvic floor rehabilitation, rather than being confounded by medical conditions (*e.g.*, obesity, chronic constipation, chronic respiratory disorders) that necessitate alternative multidisciplinary treatment pathways.

**Inclusion Criteria:** Participants were eligible if they were clinically healthy parous women aged 25–40 years with a history of vaginal delivery. This age range was selected to capture women at the peak of reproductive health while avoiding physiological and hormonal confounding associated with the perimenopausal transition.

**Exclusion Criteria:** To ensure a clinically homogeneous cohort and maximise internal validity, strict exclusion criteria were applied a priori. Based on World Health Organisation (WHO) and Rome IV standards, women with obesity (BMI ≥30 kg/m^2^), chronic cough (>8 weeks), perimenopausal period, or chronic constipation were excluded. Additionally, women currently pregnant or in the postpartum period were excluded to avoid transient pelvic floor changes.

These exclusions were protocol-driven, as this study serves as the baseline phase for a larger research program focusing on a Pelvic Floor Muscle Training (PFMT) intervention. By excluding participants with significant comorbidities, the study ensures that the identified prevalence and associations reflect pelvic floor integrity rather than general medical conditions. All screening was performed by the Principal Investigator (a Registered Nurse). While the protocol specified counselling and referral for excluded conditions, no women meeting these criteria were identified within the randomised sampling frame during screening.

### Screening process

Before enrolment, all eligible women were screened for chronic conditions that could independently influence the development of stress urinary incontinence. Information was obtained through two sources: (1) periodic health records maintained by community health workers, (2) direct participant questioning using standardised clinical definitions for common conditions by the Principal Investigator (a Registered Nurse).

1. Periodic health record Review: Official periodic health records maintained by community health workers were reviewed for documented height, weight, and pre-existing medical diagnosis. These registries provide the standard longitudinal data used for community health monitoring in this region.

2. Clinical Screening Interview: The investigator conducted face-to-face screening using standardised clinical definitions to verify current health status:

• Chronic Cough: Defined as a cough persisting for ≥ 8 weeks or a physician-diagnosed respiratory disorder (*e.g.*, asthma or COPD) (Morice et al., 2007).
• Chronic Constipation: Screened using Rome IV criteria, requiring at least two symptoms (*e.g.*, straining, hard stools, incomplete evacuation) for a minimum of three months (Aziz et al., 2020).
• Obesity: Defined as a BMI ≥ 30 kg/m^2^ based on the official health worker registries

Obesity was defined using the WHO international cutoff (BMI ≥30 kg/m^2^) to exclude clinically significant obesity as a major non-obstetric confounder (Lin & Li, 2021).

• Perimenopausal/menopausal transition: women with irregular cycles/cessation of menstruation (Harlow et al., 2012; Monteleone et al., 2018).

The screening was based on the above operational definitions, conducted through investigator-administered clinical interviews, and used solely for exclusion screening; the complete Rome IV constipation assessment and comprehensive European Respiratory Society (ERS) cough evaluation were not administered.

Height and weight information were obtained from periodic health records maintained by community health workers. In cases where height or weight records were incomplete or outdated, the respective community health worker updated the anthropometric information during routine community follow-up, or, when necessary, eligible women were contacted and requested to attend the Anganwadi centre or primary health centre for record updating before participant screening. Thus, complete height and weight data were available for all 412 participants included in the analysis. No missing BMI values were present, and BMI was calculated for all participants using the available height and weight measurements. None of the participants had BMI ≥30 kg/m^2^.

The sampling frame (*N* = 736) comprised registry-identified parous women who met age and delivery eligibility criteria. Clinical screening for exclusion conditions was conducted only among women who were randomly selected and consented to participate (*n* = 412).

Based on this assessment, none of the enrolled participants (*n* = 412) met the predefined exclusion criteria. The absence of obesity in this cohort may be attributed to the traditionally active lifestyle and dietary patterns prevalent among rural coastal women in this region; however, the investigator acknowledges that the zero prevalence of chronic cough and constipation may also reflect a degree of reporting or social-desirability bias, or limitations of retrospective registry data.

By applying these protocol-driven exclusions, the study established a clinically defined cohort, ensuring that the observed SUI prevalence reflects associations within a relatively healthy reproductive-age population rather than being influenced by major chronic comorbidities.

### Data collection instruments

#### Clinical assessment procedure of SUI with modified ICS cough stress test

Stress urinary incontinence (SUI) was objectively assessed using a modified ICS cough stress test, adapted from the ICS Educational Module. The standard ICS-Uniform Cough Stress Test is performed in the supine or lithotomy position with a retrograde-filled bladder, whereas standing assessment is described as an accessory stress test when the initial test is negative (Guralnick et al., 2018). In the present community-based study, assessment was performed sequentially in a modified supine position (knees flexed and hips abducted), followed by the standing position as a pragmatic adaptation to improve case detection under field conditions. This dual-position approach was adopted to enhance diagnostic sensitivity, as the standing position increases intra-abdominal pressure due to gravity. To ensure standardisation and minimise inter-observer variability, all clinical assessments were performed independently by the Principal Investigator, a Registered Nurse with specialised clinical training. While a single-occasion cough stress test in field conditions may be subject to transient situational variability, the use of a strict hydration protocol, standardised instructions, and dual-position testing was implemented as a quality control measure to minimise the risk of underdiagnosis.

### Standardisation of bladder volume

To approximate a comfortably filled bladder without invasive catheterisation, a standardised oral hydration protocol was employed (Guralnick et al., 2018). Following complete spontaneous voiding, participants consumed 500 mL of water, and the cough stress test was performed 30–45 min later once the participant reported a strong but comfortable desire to void. This protocol was adopted as a pragmatic field adaptation to achieve a comfortably filled bladder without catheterisation. However, bladder filling following oral hydration varies among individuals, and the actual bladder volume at the time of testing was not objectively measured. Assessments were conducted in a private, screened-off area within the community health centre or the participant’s home to ensure privacy and psychological comfort, which is essential for accurate clinical elicitation of leakage.

A positive cough stress test was interpreted as evidence of clinically detectable stress urinary incontinence; however, symptom severity, frequency, and quality-of-life impact were not assessed using validated instruments in this phase of the study.

### Development and finalisation of the obstetric risk factor assessment questionnaire

A structured questionnaire was utilised to collect data on socio-demographic characteristics (age, qualification, occupation) and obstetric risk factors (age at first delivery, parity, episiotomy, perineal tears, instrumental delivery, and delivery of neonates with a birth weight > 3.5 kg). To ensure data accuracy, these obstetric details were verified through a review of participants’ mothers’ and Child Protection cards (Thayi cards) and supplemented with structured clinical interviews.

The obstetric risk assessment questionnaire was validated by a panel of 11 experts, yielding a Scale Content Validity Index (S-CVI) of 0.91. Items were evaluated for relevance, clarity, and simplicity. Stability was established through a test-retest procedure with 10 parous women (excluded from the main study). The tool demonstrated high stability with a Pearson correlation coefficient (r) of 0.88 over a two-week interval. Face validity was further confirmed through cognitive debriefing during the pilot phase to ensure cultural appropriateness in the rural setting.

### Pre-testing of the tool

The tool was pre-tested among 10 participants to assess the feasibility and the clarity of the clinical protocol. This phase helped identify ambiguities in the questionnaire and evaluate its cultural acceptability in the community setting. The practicality of the 500 ml fluid-intake protocol and the adequacy of private screening arrangements were also assessed. Minor modifications were made following the pre-test, including rephrasing two items from obstetric questionnaire to better reflect locally understood terminology. As no major changes were required, the tool was considered feasible, and the study proceeded to the full recruitment phase.

### Study permission

Ethical approval was obtained from the Yenepoya Ethics Committee-1, Yenepoya (Deemed to be University), Mangaluru, India (Approval No: YEC-1/2021/064). Prior to enrolment, the study purpose was explained to all potential participants in their local language, and written informed consent was obtained. Confidentiality was maintained throughout the study by using anonymised codes for all data collection forms. The study adhered to the ethical standards of the institutional ethics committee and the principles of the Declaration of Helsinki. In addition, administrative permissions were obtained from the District Health Officer (DHO), Mangaluru, and the Directorate of Health and Family Welfare, Bangalore, Government of Karnataka (Ref. No: NHM/SPM/4-PART FILE/2020-21). Data was collected between September and December 2024.

### Statistical analysis

Data were analysed using IBM SPSS Statistics for Windows, Version 27.0 (IBM, Armonk, NY, USA). Descriptive statistics were used to summarise socio-demographic and obstetric characteristics, including mean ± standard deviation (SD) for continuous variables and frequencies with percentages for categorical variables.

Given the high prevalence of stress urinary incontinence (SUI) in the study population (53.4%), modified Poisson regression with robust variance was used to estimate prevalence ratios, as odds ratios from logistic regression may overestimate associations when the outcome is common.

Additionally, the raw data were internally verified by cross-referencing SUI status with age and parity distributions to ensure coding accuracy.

Maternal age, parity, and age at first delivery were included in the multivariable model as continuous variables to preserve information and maximise statistical power, avoiding arbitrary categorisation. These variables were selected based on a pre-specified conceptual framework (Fig. 1) and their established clinical relevance to pelvic floor dysfunction. Variables with extremely low frequencies were excluded from multivariable analysis to maintain model stability and prevent unreliable estimates arising from sparse data. Given the biological relationship among age, parity, and age at first delivery, adjusted estimates were interpreted cautiously within the context of the model. Statistical significance was set at *p* < 0.05.

## Results

Table 1 presents the socio-demographic characteristics of the study participants (*n* = 412). The mean age of the participants was 32.19 ± 4.48 years. Regarding educational attainment, the majority of women (59.2%, *n* = 244) had completed higher secondary education, while 20.4% (*n* = 84) held a degree or diploma, 17.5% (*n* = 72) had secondary education, and 2.9% (*n* = 12) had primary education. In terms of socio-economic status, 57.8% (*n* = 238) were classified as Below Poverty Line (BPL), and 42.2% (*n* = 174) as Above Poverty Line (APL) according to the Government of India classification. Only 1.7% (*n* = 7) of participants had prior information regarding SUI management, with healthcare workers being the primary source (*n* = 5); however, none had undergone pelvic floor muscle training or treatment. The clinical assessment of stress urinary incontinence (SUI) status revealed that 53.4% (*n* = 220) of the women exhibited symptoms, while 46.6% (*n* = 192) did not.

**Table 1:** Socio-demographic characteristics of participants.

		*N* = 412
**Sl. No.**	**Demographic proforma**	**f (%)**
**1.**	**Age in years:**		Mean ± SD = 32.19 ± 4.48 years
**2.**	**Qualification**	
	Primary	12 (2.9)
	Secondary	72 (17.5)
	Higher secondary	244 (59.2)
	Degree/diploma	84 (20.4)
**3.**	**Socioeconomic Status as per government of India**	
	APL card holder(annual income ≥1,20,000rupees)	174 (42.2)
	BPL card holder (annual income <1,20,000rupees)	238 (57.8)
**4.**	**Previous information on SUI management**	
	Yes	7 (1.7)
	NO	405 (98.3)
	**If yes, Source of Previous information (*n* = 7)**	
	Health care worker	5 (71.4)
	Friends	2 (28.6)
**5.**	**Stress urinary incontinence Status**	
	Present	220 (53.4)
	Absent	192 (46.6)

Obstetric profiles showed that the participants’ mean age at first delivery was 26.22 ± 1.87 years. Parity distribution, as shown in Table 2, indicated that 45.9% (*n* = 189) of the women had one vaginal delivery, 35.2% (*n* = 145) had two, 12.9% (*n* = 53) had three, 4.6% (*n* = 19) had four and 1.5% (*n* = 6) had 5 deliveries. Clinical histories further revealed that only 1.2% (*n* = 5) of the participants had a history of instrumental vaginal delivery or had delivered a baby weighing more than 3.5 kg. A history of episiotomy in at least one vaginal delivery was reported by all participants 100%, (*n* = 412), reflecting the localised obstetric practice in the study region during the participants’ delivery period, where episiotomy was commonly performed, particularly among primiparous women. Spontaneous perineal lacerations were infrequent, occurring in only 1.7% (*n* = 7) of cases.

**Table 2:** Obstetric profile of participants.

		*N* = 412
**Sl. No.**	**Obstetric variable**	**f (%)**
1.	**Age at first delivery:** ** Mean ± Standard deviation:**		26.22 ± 1.87 years	
2.	**Number of vaginal delivery**	
	1	189 (45.9)
	2	145 (35.2)
	3	53 (12.9)
	4	19 (4.6)
	5	6 (1.5)
3.	**History of instrumental vaginal delivery (vacuum/forceps)**	
	Yes	5 (1.2)
	No	407 (98.8)
4.	**History of delivery of a baby with a birth weight above 3.5 kg**	
	Yes	5 (1.2)
	No	407 (98.8)
5.	**Perineal trauma**	
	**a) History of episiotomy (in any previous delivery**)	
	Yes	412 (100)
	**b) Perineal laceration occurred spontaneously with or without episiotomy**	
	Yes	7 (1.7)
	No	405 (98.3)

Table 3 presents the crude and adjusted associations between maternal age and stress urinary incontinence (SUI) using modified Poisson regression with robust variance. Maternal age was significantly associated with SUI prevalence in both the crude analysis (cPR = 1.16; 95% CI [1.14–1.187]; *p* < 0.001) and the adjusted analysis (aPR = 1.198; 95% CI [1.168–1.229]; *p* < 0.001), indicating that each one-year increase in maternal age was associated with a 19.8% higher prevalence of SUI.

**Table 3:** Association of selected obstetric variables with SUI.

				*N* = 412
**Variable**	**cPR (95% CI)**	***p*-value**	**aPR (95% CI)**	***p*-value**
Maternal age	1.16 (1.14–1.187)	<0.001	1.198 (1.168–1.229)	<0.001

**Notes.**

cPR crude prevalence ratio aPR adjusted prevalence ratio CI confidence interval

Statistical significance was set at *p* < 0.05. Modified Poisson regression with robust standard errors was used to estimate prevalence ratios. This Table presents the primary model evaluating maternal age. The three-predictor multivariable model, incorporating maternal age, parity, and age at first delivery, is provided in Table S1 as a sensitivity analysis to demonstrate collinearity.

A multivariable sensitivity analysis including maternal age, parity, and age at first delivery is presented in Table S1. In this model, the adjusted estimates for parity and age at first delivery showed inverse associations, consistent with strong collinearity with maternal age; therefore, these estimates should not be interpreted as independent effects.

To further examine the robustness of the age association, a sensitivity analysis using two-year age bands demonstrated a progressive increase in SUI prevalence across the 25–40 year age range (Table S2), supporting the consistency of the association between increasing maternal age and SUI prevalence.

## Discussion

This study identified a 53.4% prevalence of SUI among 412 parous women. While this reflects a significant clinical burden, it represents a relatively healthy subset with exclusion of major non-obstetric risk factors. This prevalence is higher than estimates from mainland China (24.5%) (Li et al., 2024) but aligns with rural Jordanian data (55.1%) (Azab et al., 2021). However, when comparing these rates, it is vital to note that our findings are specific to a younger (25–40 years) and relatively healthy cohort and should not be over-extrapolated to represent the entire spectrum of rural Indian women. Compared to other Indian studies (Sharma, Khandhedia & Dave, 2022; Soni & Bindra, 2023; Rathod & Suvarna, 2024), the higher prevalence likely reflects the use of the objective modified ICS Cough Stress Test rather than self-reported questionnaires, which often underestimate leakage due to cultural normalisation. Although our data reflects objective test-positive cases rather than treatment-seeking individuals, the high prevalence underscores a significant opportunity for early intervention. According to ICS principles, once SUI is detected, early conservative management such as pelvic floor muscle training can be initiated to prevent progression to moderate or severe leakage. While the CST confirms the presence of SUI, the current study did not categorise clinical severity, which should be considered when interpreting the overall impact on the cohort.

Advancing maternal age demonstrated the strongest independent association with SUI in the adjusted model. This pattern is consistent with global evidence indicating that age-related connective tissue and pelvic floor changes increase vulnerability to incontinence (Li et al., 2024; Azab et al., 2021; Berhoğlu & Barut, 2020; Yang & Liao, 2022). Although age at first delivery and parity were associated with SUI in crude analyses, these associations attenuated and reversed direction after adjustment. This finding should be interpreted cautiously because higher parity and advancing maternal age showed substantial overlap in this cohort; notably, all women with parity ≥3 were concentrated in the older age group (31–40 years) and were SUI-positive. This distribution limited the statistical separability of maternal age and parity in the adjusted model. Therefore, the inverse association observed for parity should not be interpreted as evidence of a protective effect, but rather as a consequence of the dataset’s interdependence and structure. Within these limitations, maternal age showed the most consistent association with SUI prevalence.

Other obstetric factors, including spontaneous perineal tears, instrumental deliveries, and birth weight (>3.5 kg), were excluded from multivariable analysis due to low exposure counts. Although stress urinary incontinence is recognised as a multifactorial condition involving anatomical, neuromuscular, hormonal, and obstetric influences, the very low frequency of certain obstetric exposures in this cohort limited their inclusion in adjusted regression modelling. While previous literature has established strong associations between these specific obstetric traumas and long-term SUI (Frohlich & Kettle, 2015; Tähtinen et al., 2016), our data were insufficient for stable prevalence modelling. Notably, all participants with these specific exposures had SUI; however, due to the small sample size and potential overlap between categories, multivariable analysis was not feasible. Therefore, the adjusted findings should be interpreted within the context of the available variability rather than as a comprehensive representation of all potential obstetric determinants.

A notable finding in this cohort was the universal reporting of a history of episiotomy in at least one vaginal delivery. This likely reflects the obstetric practices prevalent in the study region during the participants’ delivery period, where episiotomy was commonly performed during institutional vaginal births, particularly among primiparous women. Because all participants reported exposure to an episiotomy at least once, the variable lacked sufficient variability to evaluate it as a potential risk factor. Nevertheless, this finding provides important insight into the obstetric care environment experienced by this population. The near-universal exposure to episiotomy observed in this cohort represents an important maternal health finding in its own right and highlights the need to promote evidence-based, selective use of episiotomy practices and to strengthen adherence to current obstetric care recommendations in maternity services.

An important public-health finding was the extremely low awareness of SUI management (1.7%), consistent with reports from other settings where stigma and limited health literacy delay care-seeking (Azab et al., 2021). In rural India, urinary leakage is often normalised as an inevitable consequence of childbirth and ageing, contributing to silence and delayed treatment (Rathod & Suvarna, 2024). The Theory of Planned Behaviour offers a useful explanatory framework, suggesting that attitudes, social norms, and perceived behavioural control influence help-seeking behaviours (Bosnjak, Ajzen & Schmidt, 2020; Coughlin et al., 2020; Mohd Tohit & Haque, 2024).

Beyond individual influences, health-system barriers—including a lack of routine postnatal screening, limited referral pathways, and inadequate privacy at primary centres—contribute to persistent untreated morbidity. Current evidence from the most recent National Family Health Survey (NFHS-5) suggests that despite an overall increase in institutional births, significant geographical disparities remain, with certain rural regions characterised by low coverage of comprehensive maternal services (Bajpai et al., 2025). These structural gaps help explain the low awareness observed in our cohort and underscore the need to integrate SUI counselling and frontline health worker engagement into routine maternal care. A practical approach may include training frontline community health workers such as Accredited Social Health Activists (ASHAs) to perform simple symptom-based screening for urinary incontinence during postnatal home visits, provide basic pelvic floor health education, and facilitate referral of symptomatic women to appropriate healthcare services.

While this study focuses on a relatively healthy, non-obese cohort, these findings provide a critical foundational baseline for specific subgroups within national health strategies. The observed prevalence (53.4%) in this younger, healthy population suggests a significant early burden of SUI, though it may not be directly generalised to the broader, more medically diverse population of rural women who may present with additional risk factors such as obesity or chronic respiratory conditions. Identifying such a high prevalence (53.4%) in a younger, healthy population suggests that SUI begins early in the reproductive trajectory, long before the onset of age-related comorbidities or obesity. This evidence supports a tiered national strategy where early pelvic floor education is integrated into routine postnatal care for all women, while also serving as a justification for intensified screening and resources for older, medically comorbid, or obese populations who likely carry an even higher burden of disease.

## Limitations

This cross-sectional study permits identification of associations but not causal inference. Although the modified ICS Cough Stress Test provides objective clinical confirmation of leakage, the absence of validated symptom severity or quality-of-life measures limits interpretation of the functional burden associated with the observed prevalence. Furthermore, as symptom severity was not categorized, the 53.4% prevalence reflects test-positive SUI rather than the proportion of moderate-to-severe or treatment-seeking cases. The cough stress test was performed by the same investigator who collected obstetric information, and assessor blinding to exposure status was not feasible in this community-based setting. Although standardised instructions and a predefined clinical protocol were followed to minimise variability, the possibility of observer bias cannot be completely excluded.

The study population had limited representation of women with higher BMI and comorbid conditions; therefore, the findings should not be over-generalised to the broader and medically diverse population of rural Indian women who may have different risk profiles.

The sample size was primarily powered for prevalence estimation using a hospital-based prevalence estimate; the study may have been underpowered to detect significant independent associations for rare obstetric exposures, such as macrosomia or instrumental delivery. As hospital-based prevalence estimates may overestimate baseline SUI prevalence compared with community-based populations, this should be considered when interpreting the precision and generalizability of the prevalence estimate. Screening for predefined exclusion criteria was conducted through participant interviews supported by community health records rather than specialist clinical assessment, which may have led to under-identification of some exclusion criteria. In addition, the low frequency of certain obstetric exposures (*e.g.*, macrosomia, instrumental delivery, perineal trauma) constrained multivariable modelling, and the interrelatedness between maternal age and reproductive factors may have influenced adjusted estimates and should be considered when interpreting the adjusted associations. Furthermore, reliance on registry data and self-reported obstetric history may introduce information bias. Awareness of SUI management was assessed with a single dichotomous item rather than a validated awareness or health literacy instrument. Therefore, the low proportion reporting prior awareness (1.7%) should be interpreted cautiously, as responses obtained through face-to-face interviews with a healthcare provider may have been influenced by recall or social-desirability bias.

## Conclusion

This community-based study identified a high prevalence of stress urinary incontinence (53.4%) among a specifically screened cohort of relatively healthy parous women aged 25–40 years in rural coastal Karnataka. Despite this burden, awareness of SUI management was extremely limited (1.7%), indicating substantial normalisation and unmet educational needs.

In the final multivariable model, maternal age was the only factor independently associated with SUI. Although parity and age at first delivery were associated with SUI in unadjusted analyses, these associations did not remain significant after adjustment for maternal age. These findings represent statistical associations and should not be interpreted as causal.

Although restricted to a relatively healthy reproductive-age subset, the results underscore the need to strengthen pelvic health awareness and consider integration of routine SUI screening within primary maternal healthcare services. Future research is warranted to determine the prevalence and risk factors in older or medically comorbid populations to build a more comprehensive national profile of SUI.

##  Supplemental Information

10.7717/peerj.21722/supp-1 Supplemental Information 1 Anonymized dataset of socio-demographic, obstetric variables, and cough stress test results from 412 parous women in rural Mangaluru raw, anonymized participant-level data used in the analysis of stress urinary incontinence prevalence and obstetric risk factors. Variables include age, education, socioeconomic status, age at first delivery, number of vaginal deliveries, obstetric history, and SUI status (cough stress test results).

10.7717/peerj.21722/supp-2 Supplemental Information 2 STROBE Checklist

10.7717/peerj.21722/supp-3 Supplemental Information 3 Adjusted Modified Poisson Regression SPSS Output SPSS output containing the adjusted modified Poisson regression analysis assessing the association between obstetric risk factors and stress urinary incontinence after adjustment for covariates.

10.7717/peerj.21722/supp-4 Supplemental Information 4 Crude Modified Poisson Regression for Maternal Age SPSS output presenting the crude modified Poisson regression analysis evaluating the association between maternal age and stress urinary incontinence.

10.7717/peerj.21722/supp-5 Supplemental Information 5 Crude Modified Poisson Regression: Age at First Delivery SPSS output presenting the crude modified Poisson regression analysis evaluating the association between age at first delivery and stress urinary incontinence.

10.7717/peerj.21722/supp-6 Supplemental Information 6 Data collection tool the tool includes demographic proforma, obstetric proforma and cough stress test

10.7717/peerj.21722/supp-7 Supplemental Information 7 Socio-demographic analysis SPSS Output SPSS output containing the descriptive statistical analyses used to generate Table 1- participants’ sociodemographic characteristics.

10.7717/peerj.21722/supp-8 Supplemental Information 8 Obstetric variables analysis SPSS Output SPSS output containing the descriptive statistical analyses used to generate Table 2- participants’ obstetric characteristics.

10.7717/peerj.21722/supp-9 Supplemental Information 9 Crude and adjusted modified Poisson regression analysis for factors associated with stress urinary incontinence

10.7717/peerj.21722/supp-10 Supplemental Information 10 Age-specific prevalence of stress urinary incontinence using two-year age bands

10.7717/peerj.21722/supp-11 Supplemental Information 11 English code book for spss crude and adjusted output

10.7717/peerj.21722/supp-12 Supplemental Information 12 Crude Modified Poisson Regression: Parity SPSS output presenting the crude modified Poisson regression analysis evaluating the association between parity and stress urinary incontinence.

## Additional Information and Declarations

### Field Study Permissions

The following information was supplied relating to field study approvals (*i.e.*, approving body and any reference numbers): The study was approved by the Yenepoya Ethics Committee-1, Yenepoya (Deemed to be University), Mangaluru, India (Approval No: YEC-1/2021/064). In addition, administrative permissions for community-based data collection were obtained from the District Health Officer (DHO), Mangaluru, and from the Directorate of Health and Family Welfare, Bangalore, Government of Karnataka (Ref. No: NHM/SPM/4-PART FILE/2020-21). No additional field permits were required.

### Data Availability

The following information was supplied regarding data availability: The data and code are available in the Supplemental Files.
