# Supplementary material for: Prevalence and obstetric risk factors of stress urinary incontinence in parous women: a cross-sectional study in rural areas of Mangaluru, India
Source: PeerJ. 2026 Sep 8;14:e21722. doi: 10.7717/peerj.21722 (PMC13584930; doi:10.7717/peerj.21722)
Supplement: Supplementary file 1 [file peerj-14-21722-s004.pdf]

```
* Generalized Linear Models.
GENLIN SUI_score WITH age
  /MODEL age INTERCEPT=YES
 DISTRIBUTION=POISSON LINK=LOG
  /CRITERIA METHOD=FISHER(1) SCALE=1 COVB=ROBUST MAXITERATIONS=100 MAXSTEPHALVING=5
    PCONVERGE=1E-006(ABSOLUTE) SINGULAR=1E-012 ANALYSISTYPE=3(WALD) CILEVEL=95 CITYPE=WAL
D
    LIKELIHOOD=FULL
  /MISSING CLASSMISSING=EXCLUDE
  /PRINT CPS DESCRIPTIVES MODELINFO FIT SUMMARY SOLUTION (EXPONENTIATED).
```

## Generalized Linear Models

### Model Information

| | |
|---|---|
| Dependent Variable | SUI_score |
| Probability Distribution | Poisson |
| Link Function | Log |

### Case Processing Summary

| | N | Percent |
|---|---|---|
| Included | 412 | 100.0% |
| Excluded | 0 | 0.0% |
| Total | 412 | 100.0% |

### Continuous Variable Information

| | | N | Minimum | Maximum | Mean | Std. Deviation |
|---|---|---|---|---|---|---|
| Dependent Variable | SUI_score | 412 | 0 | 1 | .53 | .499 |
| Covariate | age | 412 | 25 | 40 | 32.19 | 4.484 |

## Goodness of Fit[a]

| | Value | df | Value/df |
|---|---|---|---|
| Deviance | 180.180 | 410 | .439 |
| Scaled Deviance | 180.180 | 410 | |
| Pearson Chi-Square | 166.460 | 410 | .406 |
| Scaled Pearson Chi-Square | 166.460 | 410 | |
| Log Likelihood[b] | -310.090 | | |
| Akaike's Information Criterion (AIC) | 624.180 | | |
| Finite Sample Corrected AIC (AICC) | 624.210 | | |
| Bayesian Information Criterion (BIC) | 632.222 | | |
| Consistent AIC (CAIC) | 634.222 | | |

Dependent Variable: SUI_score
Model: (Intercept), age[a]

a. Information criteria are in smaller-is-better form.

b. The full log likelihood function is displayed and used in computing information criteria.

## Omnibus Test[a]

| Likelihood Ratio Chi-Square | df | Sig. |
|---|---|---|
| 95.874 | 1 | .000 |

Dependent Variable: SUI_score
Model: (Intercept), age[a]

a. Compares the fitted model against the intercept-only model.

## Tests of Model Effects

| | Type III | | |
|---|---|---|---|
| Source | Wald Chi-Square | df | Sig. |
| (Intercept) | 246.079 | 1 | .000 |
| age | 244.653 | 1 | .000 |

Dependent Variable: SUI_score
Model: (Intercept), age

**Parameter Estimates**

| Parameter | B | Std. Error | 95% Wald Confidence Interval | | Hypothesis Test | |
|---|---|---|---|---|---|---|
| | | | Lower | Upper | Wald Chi-Square | df |
| (Intercept) | -5.750 | .3666 | -6.469 | -5.032 | 246.079 | 1 |
| age | .152 | .0097 | .133 | .171 | 244.653 | 1 |
| (Scale) | 1[a] | | | | | |

**Parameter Estimates**

| Parameter | Hypothesis Test | Exp(B) | 95% Wald Confidence Interval for Exp(B) | |
|---|---|---|---|---|
| | Sig. | | Lower | Upper |
| (Intercept) | .000 | .003 | .002 | .007 |
| age | .000 | 1.164 | 1.142 | 1.187 |
| (Scale) | | | | |

Dependent Variable: SUI_score
Model: (Intercept), age

a. Fixed at the displayed value.
