# Supplementary material for: Prevalence and obstetric risk factors of stress urinary incontinence in parous women: a cross-sectional study in rural areas of Mangaluru, India
Source: PeerJ. 2026 Sep 8;14:e21722. doi: 10.7717/peerj.21722 (PMC13584930; doi:10.7717/peerj.21722)
Supplement: Supplementary file 2 [file peerj-14-21722-s005.pdf]

```
* Generalized Linear Models.
GENLIN SUI_score WITH age_at_first_delivery
  /MODEL age_at_first_delivery INTERCEPT=YES
 DISTRIBUTION=POISSON LINK=LOG
  /CRITERIA METHOD=FISHER(1) SCALE=1 COVB=ROBUST MAXITERATIONS=100 MAXSTEPHALVING=5
    PCONVERGE=1E-006(ABSOLUTE) SINGULAR=1E-012 ANALYSISTYPE=3(WALD) CILEVEL=95 CITYPE=WAL
D
    LIKELIHOOD=FULL
  /MISSING CLASSMISSING=EXCLUDE
  /PRINT CPS DESCRIPTIVES MODELINFO FIT SUMMARY SOLUTION (EXPONENTIATED).
```

Generalized Linear Models

Model Information

Dependent Variable	SUI_score
Probability Distribution	Poisson
Link Function	Log

Case Processing Summary

	N	Percent
Included	412	100.0%
Excluded	0	0.0%
Total	412	100.0%

Continuous Variable Information

		N	Minimum	Maximum	Mean
Dependent Variable	SUI_score	412	0	1	.53
Covariate	age_at_first_delivery	412	22	35	26.22

Continuous Variable Information

		Std. Deviation
Dependent Variable	SUI_score	.499
Covariate	age_at_first_delivery	1.871

Goodness of Fit[a]

	Value	df	Value/df
Deviance	271.459	410	.662
Scaled Deviance	271.459	410	
Pearson Chi-Square	191.565	410	.467
Scaled Pearson Chi-Square	191.565	410	
Log Likelihood[b]	-355.730		
Akaike's Information Criterion (AIC)	715.459		
Finite Sample Corrected AIC (AICC)	715.488		
Bayesian Information Criterion (BIC)	723.501		
Consistent AIC (CAIC)	725.501		

Dependent Variable: SUI_score
Model: (Intercept), age_at_first_delivery[a]

a. Information criteria are in smaller-is-better form.

b. The full log likelihood function is displayed and used in computing information criteria.

Omnibus Test[a]

Likelihood Ratio Chi-Square	df	Sig.
4.595	1	.032

Dependent Variable: SUI_score
Model: (Intercept), age_at_first_delivery[a]

a. Compares the fitted model against the intercept-only model.

Tests of Model Effects

Source	Type III		
	Wald Chi-Square	df	Sig.
(Intercept)	23.899	1	.000
age_at_first_delivery	14.346	1	.000

Dependent Variable: SUI_score
Model: (Intercept), age_at_first_delivery

Parameter Estimates

Parameter	B	Std. Error	95% Wald Confidence Interval		Hypothesis Test	
			Lower	Upper	Wald Chi-Square	df
(Intercept)	-2.564	.5245	-3.592	-1.536	23.899	1
age_at_first_delivery	.073	.0194	.035	.112	14.346	1
(Scale)	1[a]					

Parameter Estimates

Parameter	Hypothesis Test	Exp(B)	95% Wald Confidence Interval for Exp(B)	
	Sig.		Lower	Upper
(Intercept)	.000	.077	.028	.215
age_at_first_delivery	.000	1.076	1.036	1.118
(Scale)				

Dependent Variable: SUI_score
Model: (Intercept), age_at_first_delivery

a. Fixed at the displayed value.
