# Supplementary material for: Prevalence and obstetric risk factors of stress urinary incontinence in parous women: a cross-sectional study in rural areas of Mangaluru, India
Source: PeerJ. 2026 Sep 8;14:e21722. doi: 10.7717/peerj.21722 (PMC13584930; doi:10.7717/peerj.21722)
Supplement: Supplementary file 3 [file peerj-14-21722-s012.pdf]

```
* Generalized Linear Models.
GENLIN SUI_score WITH vag_delivery_no
  /MODEL vag_delivery_no INTERCEPT=YES
 DISTRIBUTION=POISSON LINK=LOG
  /CRITERIA METHOD=FISHER(1) SCALE=1 COVB=ROBUST MAXITERATIONS=100 MAXSTEPHALVING=5
    PCONVERGE=1E-006(ABSOLUTE) SINGULAR=1E-012 ANALYSISTYPE=3(WALD) CILEVEL=95 CITYPE=WAL
D
    LIKELIHOOD=FULL
  /MISSING CLASSMISSING=EXCLUDE
  /PRINT CPS DESCRIPTIVES MODELINFO FIT SUMMARY SOLUTION (EXPONENTIATED).
```

Generalized Linear Models

Model Information

Dependent Variable	SUI_score
Probability Distribution	Poisson
Link Function	Log

Case Processing Summary

	N	Percent
Included	412	100.0%
Excluded	0	0.0%
Total	412	100.0%

Continuous Variable Information

		N	Minimum	Maximum	Mean	Std. Deviation
Dependent Variable	SUI_score	412	0	1	.53	.499
Covariate	vag_delivery_no	412	1	5	1.81	.931

Goodness of Fit[a]

	Value	df	Value/df
Deviance	239.409	410	.584
Scaled Deviance	239.409	410	
Pearson Chi-Square	187.428	410	.457
Scaled Pearson Chi-Square	187.428	410	
Log Likelihood[b]	-339.705		
Akaike's Information Criterion (AIC)	683.409		
Finite Sample Corrected AIC (AICC)	683.438		
Bayesian Information Criterion (BIC)	691.451		
Consistent AIC (CAIC)	693.451		

Dependent Variable: SUI_score
Model: (Intercept), vag_delivery_no[a]

a. Information criteria are in smaller-is-better form.

b. The full log likelihood function is displayed and used in computing information criteria.

Omnibus Test[a]

Likelihood Ratio Chi-Square	df	Sig.
36.645	1	.000

Dependent Variable: SUI_score
Model: (Intercept), vag_delivery_no[a]

a. Compares the fitted model against the intercept-only model.

Tests of Model Effects

	Type III		
Source	Wald Chi-Square	df	Sig.
(Intercept)	181.009	1	.000
vag_delivery_no	129.836	1	.000

Dependent Variable: SUI_score
Model: (Intercept), vag_delivery_no

Parameter Estimates

Parameter	B	Std. Error	95% Wald Confidence Interval		Hypothesis Test	
			Lower	Upper	Wald Chi-Square	df
(Intercept)	-1.394	.1036	-1.597	-1.191	181.009	1
vag_delivery_no	.384	.0337	.318	.450	129.836	1
(Scale)	1[a]					

Parameter Estimates

Parameter	Hypothesis Test		95% Wald Confidence Interval for Exp(B)	
	Sig.	Exp(B)	Lower	Upper
(Intercept)	.000	.248	.202	.304
vag_delivery_no	.000	1.468	1.374	1.568
(Scale)				

Dependent Variable: SUI_score
Model: (Intercept), vag_delivery_no

a. Fixed at the displayed value.
