# Supplementary material for: Prevalence and obstetric risk factors of stress urinary incontinence in parous women: a cross-sectional study in rural areas of Mangaluru, India
Source: PeerJ. 2026 Sep 8;14:e21722. doi: 10.7717/peerj.21722 (PMC13584930; doi:10.7717/peerj.21722)
Supplement: Supplementary file 4 [file peerj-14-21722-s006.docx]

**TOOL FOR DATA COLLECTION**

**Instructions: kindly place a tick mark(**✓) **in the box against the options that you feel is more suitable to you.**

**Code:**

**PART I: DEMOGRAPHIC PROFORMA**

**Instructions: We would like to know your demographic Performa and how much** they **affect you? From the list below choose only those that you have at present. Leave out those that don’t apply to you. Please tick any one.**

**1**. Age in years_________________

2. qualification

A. No formal education	[	]
B. primary	[	]
C. Secondary	[	]
D. Higher secondary	[	]
E. Degree/Diploma	[	]
F. Postgraduation and above	[	]

3. Socio economic status

A. APL card holder	[	]

B. BPL card Holder	[	]

4. Previous information

A) Yes	[	]

B) No	[	]

Sources of information

A. Relatives/friends/neighbors	[	]
B. media	[	]
C. From health care workers	[	]
D. Journals	[	]

**Part II : Obstetric Data assessment proforma**

1. Age at first delivery_________________years

2. Number of deliveries________________

3. History of instrumental delivery like vacuum/forceps:

A. YES	[	]

B. NO	[	]

4. Birth weight of the baby above 3.5 kg if any___________

A. YES	[	]

B. NO	[	]

5. Did you undergo episiotomy (planned surgical incision on perineum) during any of the previous delivery?

A. YES	[	]

B. NO	[	]

6. Apart from episiotomy did you suffer any perineal tear/ laceration/ injury or extension of episiotomy?

A. YES	[	]

B. NO	[	]

**Tool II: Cough stress test: Assessed by investigator**

**(description in manuscript)**
