# Supplementary material for: Prevalence and obstetric risk factors of stress urinary incontinence in parous women: a cross-sectional study in rural areas of Mangaluru, India
Source: PeerJ. 2026 Sep 8;14:e21722. doi: 10.7717/peerj.21722 (PMC13584930; doi:10.7717/peerj.21722)
Supplement: Supplementary file 5 [file peerj-14-21722-s009.docx]

**Results**

**Table S1. Crude and adjusted modified Poisson regression analysis for factors associated with stress urinary incontinence**

**N = 412**

| **Variable** | **cPR (95% CI)** | **p-value** | **aPR (95% CI)** | **p-value** |
| --- | --- | --- | --- | --- |
| Age (years) | 1.16 (1.14–1.187) | <0.001 | 1.198 (1.168–1.229) | <0.001 |
| Age at first delivery (years) | 1.076 (1.036–1.118) | <0.001 | 0.959 (0.92–0.997) | 0.035 |
| Parity | 1.468 (1.37–1.568) | <0.001 | 0.85 (0.777–0.93) | <0.001 |

***cPR:*** *Crude prevalence ratio;* ***aPR:*** *Adjusted prevalence ratio;* ***CI:*** *Confidence interval. Statistical significance was set at p < 0.05. All variables were mutually adjusted in the model using modified Poisson regression with robust standard errors.*

***Note:*** *The adjusted estimates for parity and age at first delivery should be interpreted cautiously, as these variables are strongly correlated with maternal age. The observed inverse associations likely reflect adjustment for maternal age and should be considered model-dependent adjusted associations rather than independent causal effects.*
