# Supplementary material for: Prevalence and obstetric risk factors of stress urinary incontinence in parous women: a cross-sectional study in rural areas of Mangaluru, India
Source: PeerJ. 2026 Sep 8;14:e21722. doi: 10.7717/peerj.21722 (PMC13584930; doi:10.7717/peerj.21722)
Supplement: Supplementary file 6 [file peerj-14-21722-s010.docx]

**Table S2. Age-specific prevalence of stress urinary incontinence**

**N = 412**

| **Age group (years)** | **Total participants**  **(n)** | **SUI-positive participants**  **(n)** | **SUI prevalence**  **(%)** |
| --- | --- | --- | --- |
| 25–26 | 44 | 0 | 0.0 |
| 27–28 | 67 | 11 | 16.4 |
| 29–30 | 55 | 17 | 30.9 |
| 31–32 | 56 | 22 | 39.3 |
| 33–34 | 54 | 39 | 72.2 |
| 35–36 | 45 | 41 | 91.1 |
| 37–38 | 43 | 42 | 97.7 |
| 39–40 | 48 | 48 | 100.0 |

*SUI: Stress urinary incontinence. Values are presented as number (n) and percentage (%).*

***Note:*** *SUI prevalence (%) was calculated as the proportion of SUI-positive participants within each age group. The high prevalence observed in older age strata reflects objective clinical test-positivity on the modified cough stress test within this parous cohort, rather than severe or treatment-seeking clinical morbidity.*
