# Supplementary material for: Prevalence and obstetric risk factors of stress urinary incontinence in parous women: a cross-sectional study in rural areas of Mangaluru, India
Source: PeerJ. 2026 Sep 8;14:e21722. doi: 10.7717/peerj.21722 (PMC13584930; doi:10.7717/peerj.21722)
Supplement: Supplementary file 7 [file peerj-14-21722-s011.docx]

**English-Language Codebook: Crude Maternal Age Analysis**

crude age SPSS output

**Purpose.** This English-language codebook defines the SPSS variable names used in the corresponding statistical output and provides reader-friendly labels, definitions, coding, and measurement units.

# Variables included in the analysis

| **SPSS variable** | **English variable label** | **Definition** | **Coding / unit** | **Observed range** |
| --- | --- | --- | --- | --- |
| SUI_score | Stress urinary incontinence status | Binary indicator of SUI status based on the cough stress test used in the study. | 0 = No SUI; 1 = SUI | 0–1 |
| age | Maternal age | Age of the participant at the time of assessment. | Years | 25–40 years |

# Dataset information

**•** Number of participants included: 412.

**•** Excluded cases due to missing values in the analysed variables: 0.

**•** Analysis represented by this output: Crude modified Poisson regression analysis of maternal age associated with stress urinary incontinence.

**•** The statistical output uses a Poisson distribution with a log link and robust covariance estimates.

**English-Language Codebook: Crude Age at First Delivery Analysis**

crude age at first delivery SPSS output

# Variables included in the analysis

| **SPSS variable** | **English variable label** | **Definition** | **Coding / unit** | **Observed range** |
| --- | --- | --- | --- | --- |
| SUI_score | Stress urinary incontinence status | Binary indicator of SUI status based on the cough stress test used in the study. | 0 = No SUI; 1 = SUI | 0–1 |
| age_at_first_delivery | Age at first delivery | Age of the participant at her first delivery. | Years | 22–35 years |

# Dataset information

**•** Number of participants included: 412.

**•** Excluded cases due to missing values in the analysed variables: 0.

**•** Analysis represented by this output: Crude modified Poisson regression analysis of age at first delivery associated with stress urinary incontinence.

**•** The statistical output uses a Poisson distribution with a log link and robust covariance estimates.

**English-Language Codebook: Crude Parity Analysis**

crude parity SPSS output

# Variables included in the analysis

| **SPSS variable** | **English variable label** | **Definition** | **Coding / unit** | **Observed range** |
| --- | --- | --- | --- | --- |
| SUI_score | Stress urinary incontinence status | Binary indicator of SUI status based on the cough stress test used in the study. | 0 = No SUI; 1 = SUI | 0–1 |
| vag_delivery_no | Number of vaginal deliveries (parity) | Total number of vaginal deliveries reported by the participant. | Number of deliveries | 1–5 deliveries |

# Dataset information

**•** Number of participants included: 412.

**•** Excluded cases due to missing values in the analysed variables: 0.

**•** Analysis represented by this output: Crude modified Poisson regression analysis of number of vaginal

deliveries (parity) associated with stress urinary incontinence.

**•** The statistical output uses a Poisson distribution with a log link and robust covariance estimates.

**English-Language Codebook – Adjusted Prevalence Ratio Analysis**

Variables used in the adjusted modified Poisson regression model

| **Dataset / analysis sample** | 412 participants |
| --- | --- |
| **Outcome variable** | Stress urinary incontinence (SUI) status |
| **Model** | Poisson regression with log link and robust covariance estimator |
| **Missing/excluded cases** | 0 excluded; 412 included |

# Variable definitions and coding

| **SPSS variable** | **English variable label** | **Definition** | **Coding / unit** | **Observed range** | **N** |
| --- | --- | --- | --- | --- | --- |
| SUI_score | Stress urinary incontinence status | Binary outcome indicating whether stress urinary incontinence was present based on the study assessment. | 0 = No SUI; 1 = SUI | 0–1 | 412 |
| age | Maternal age | Age of the participant at the time of assessment. | Years | 25–40 | 412 |
| age_at_first_delivery | Age at first delivery | Age of the participant at the time of her first delivery. | Years | 22–35 | 412 |
| vag_delivery_no | Number of vaginal deliveries (parity) | Total number of vaginal deliveries reported by the participant. | Number of vaginal deliveries | 1–5 | 412 |

**Analysis note:** The adjusted model included maternal age, age at first delivery, and number of vaginal deliveries (parity) simultaneously. The dependent variable was SUI_score. The SPSS output reports 412 included observations and no excluded observations.

**Key adjusted estimates (for reference):** maternal age aPR 1.198 (95% CI 1.168–1.229; p<0.001); age at first delivery aPR 0.959 (95% CI 0.923–0.997; p=0.035); number of vaginal deliveries aPR 0.851 (95% CI 0.777–0.931; p<0.001).

Please note that the SPSS output files were requested by the reviewer for verification, not for publication.

Also, this English codebook is for the technical team, not for publication
