# Supplementary material for: Prevalence and obstetric risk factors of stress urinary incontinence in parous women: a cross-sectional study in rural areas of Mangaluru, India
Source: PeerJ. 2026 Sep 8;14:e21722. doi: 10.7717/peerj.21722 (PMC13584930; doi:10.7717/peerj.21722)
Supplement: Supplementary file 8 [file peerj-14-21722-s002.doc]

STROBE Statement—checklist of items that should be included in reports of observational studies

|  | Item No | Recommendation | Reported (Yes/No) | Location in manuscript (page/section) |
| --- | --- | --- | --- | --- |
| **Title and abstract** | 1 | (*a*) Indicate the study’s design with a commonly used term in the title or the abstract | Yes | Page 1, Title – “…A cross-sectional study” |
|  |  | (*b*) Provide in the abstract an informative and balanced summary of what was done and what was found | Yes | Page 1, Abstract – Background, Methods, Results, Conclusion |
| Introduction | | |  |  |
| Background/rationale | 2 | Explain the scientific background and rationale for the investigation being reported | Yes | Page 2–3, Introduction – Background/rationale section |
| Objectives | 3 | State-specific objectives, including any prespecified hypotheses | Yes | Page 3, Introduction – Final paragraph: "This study aimed to estimate the prevalence of stress urinary incontinence and identify its associated obstetric and biological risk factors among parous women aged 25–40 years in rural Mangaluru, India. |
| Methods | | |  |  |
| Study design | 4 | Present key elements of study design early in the paper | Yes | Page 3, Methods – Community-based cross-sectional study. |
| Setting | 5 | Describe the setting, locations, and relevant dates, including periods of recruitment, exposure, follow-up, and data collection | Yes | Page 3 and 4, Methods – Setting: Ullal Taluk, rural Mangaluru; Page 7-recruitment period described |
| Participants | 6 | (*a*) *Cohort study*—Give the eligibility criteria, and the sources and methods of selection of participants. Describe methods of follow-up  *Case-control study*—Give the eligibility criteria, and the sources and methods of case ascertainment and control selection. Give the rationale for the choice of cases and controls  *Cross-sectional study*—Give the eligibility criteria, and the sources and methods of selection of participants |  | Pages 4–7, Methods (Study Setting and Participant Selection; Eligibility Criteria; Screening Process); Figure 2  Eligibility criteria, sampling frame (N=736), proportionate stratified random sampling across seven rural wards, simple random selection within strata, sequential replacement of non-participants (n=7), inclusion/exclusion criteria, screening for predefined exclusion criteria and participant flow are fully described. |
|  |  | (*b*) *Cohort study*—For matched studies, give matching criteria and number of exposed and unexposed  *Case-control study*—For matched studies, give matching criteria and the number of controls per case |  |  |
| Variables | 7 | Clearly define all outcomes, exposures, predictors, potential confounders, and effect modifiers. Give diagnostic criteria, if applicable | Yes | Page 5-7, Methods and materials – Outcome (SUI via cough stress test); exposures/predictors (obstetric, demographic); confounders noted |
| Data sources/ measurement | 8* | For each variable of interest, give sources of data and details of methods of assessment (measurement). Describe comparability of assessment methods if there is more than one group | *Yes* | Page 7, Methods and materials– Structured questionnaire and modified ICS Cough Stress Test; validated by experts |
| Bias | 9 | Describe any efforts to address potential sources of bias | Yes | Page 5-7, Methods and materials – Standardised cough stress test, validated tool, exclusion of chronic conditions |
| Study size | 10 | Explain how the study size was arrived at | Yes | Page 3, Methods (Sample size and sampling) - Single proportion formula based on expected prevalence (55.5%), DEFF of 1.05, and non-response adjustment. |
| Quantitative variables | 11 | Explain how quantitative variables were handled in the analyses. If applicable, describe which groupings were chosen and why | Yes | Pages 8–9, Statistical Analysis – Maternal age, parity and age at first delivery were analysed as continuous variables in the regression model. Descriptive age categories with two-year age bands shown in Supplementary Table S2 for sensitivity analysis. |
| Statistical methods | 12 | (*a*) Describe all statistical methods, including those used to control for confounding | Yes | 12 a) Pages 8–9, Statistical Analysis – Modified Poisson regression with robust variance, descriptive statistics, continuous-variable modelling, and confounder adjustment based on the conceptual framework. |
|  |  | (*b*) Describe any methods used to examine subgroups and interactions | Yes | 12 b) Pages 8–9 Statistical Analysis and Results; Supplementary Tables S1 and S2 |
|  |  | (*c*) Explain how missing data were addressed | Yes | 12 c) Pages 5–7 (Screening Process); Page 8 (Statistical Analysis)  No missing data were present for analysed variables. All 412 enrolled participants completed interviews and clinical assessment; complete anthropometric data were available for BMI calculation. |
|  |  | (*d*) *Cohort study*—If applicable, explain how loss to follow-up was addressed  *Case-control study*—If applicable, explain how matching of cases and controls was addressed  *Cross-sectional study*—If applicable, describe analytical methods taking account of sampling strategy | Yes | 12d) Pages 4–5 Sample Selection  Analyses accounted for the proportionate stratified sampling design |
|  |  | (*e*) Describe any sensitivity analyses | Yes | 12 e) Results Page 9; Supplementary Tables S1–S2.  Sensitivity analyses included a full multivariable model and age-specific prevalence analyses demonstrating robustness of the maternal-age association. |

| Results | | | Reported (Yes/No) | Location in manuscript (page/section) |
| --- | --- | --- | --- | --- |
| Participants | 13* | (a) Report numbers of individuals at each stage of study—eg numbers potentially eligible, examined for eligibility, confirmed eligible, included in the study, completing follow-up, and analysed | yes | Pages 4–8, Methods; Figure 2; Results (Page 8). Sampling frame (N=736), women approached (N=419), declined (n=7), enrolled and analysed (N=412), with no enrolled participants meeting predefined exclusion criteria. |
|  |  | (b) Give reasons for non-participation at each stage | Yes | Figure 2 |
|  |  | (c) Consider use of a flow diagram | yes | Figure 2 |
| Descriptive data | 14* | (a) Give characteristics of study participants (eg demographic, clinical, social) and information on exposures and potential confounders | Yes  Yes | Page 8, Results – Table 1 (demographics), Table 2 (obstetric); no missing data reported  Pages 5–8 Methods and Results  No missing data were present for analysed variables. |
|  |  | (b) Indicate number of participants with missing data for each variable of interest |  |  |
|  |  | (c) *Cohort study*—Summarise follow-up time (eg, average and total amount) |  |  |
| Outcome data | 15* | *Cohort study*—Report numbers of outcome events or summary measures over time |  |  |
|  |  | *Case-control study—*Report numbers in each exposure category, or summary measures of exposure |  |  |
|  |  | *Cross-sectional study—*Report numbers of outcome events or summary measures | *yes* | Page 8-9, Results – Table 1-3 |
| Main results | 16 | (*a*) Give unadjusted estimates and, if applicable, confounder-adjusted estimates and their precision (eg, 95% confidence interval). Make clear which confounders were adjusted for and why they were included | Yes  Yes | Page 9 Results – Table 3  Tables 1–3  Continuous variables were analysed; descriptive age categories were reported in sensitivity analyses using two-year age bands (Supplementary Table S2). |
|  |  | (*b*) Report category boundaries when continuous variables were categorized |  |  |
|  |  | (*c*) If relevant, consider translating estimates of relative risk into absolute risk for a meaningful time period |  |  |
| Other analyses | 17 | Report other analyses done—eg analyses of subgroups and interactions, and sensitivity analyses | yes | Page 9, Results; Supplementary Tables S1 and S2 – Sensitivity analyses included the full multivariable model and age-specific prevalence analysis. |
| Discussion | | |  |  |
| Key results | 18 | Summarise key results with reference to study objectives | Yes | Page 9-11, Discussion |
| Limitations | 19 | Discuss limitations of the study, taking into account sources of potential bias or imprecision. Discuss both direction and magnitude of any potential bias | Yes | Limitations (Pages 11–12)  Limitations include the cross-sectional design (no causal inference), lack of symptom severity and quality-of-life assessment, absence of assessor blinding, restricted healthy study population limiting generalisability, possible information and recall bias, limited power for rare obstetric exposures, potential collinearity among reproductive variables, and possible under-identification of exclusion conditions. |
| Interpretation | 20 | Give a cautious overall interpretation of results considering objectives, limitations, multiplicity of analyses, results from similar studies, and other relevant evidence | Yes | Page 9-11, Discussion – Compared with international/Indian studies; cautious interpretation |
| Generalisability | 21 | Discuss the generalisability (external validity) of the study results | Yes | Pages 9–12, Discussion and Conclusion – Generalisability discussed in relation to the relatively healthy reproductive-age rural population and acknowledged as limited for medically diverse populations. |
| Other information | | |  |  |
| Funding | 22 | Give the source of funding and the role of the funders for the present study and, if applicable, for the original study on which the present article is based | Yes | Page 12, Funding –No specific grant obtained |

*Give information separately for cases and controls in case-control studies and, if applicable, for exposed and unexposed groups in cohort and cross-sectional studies.

**Note:** An Explanation and Elaboration article discusses each checklist item and gives methodological background and published examples of transparent reporting. The STROBE checklist is best used in conjunction with this article (freely available on the Web sites of PLoS Medicine at http://www.plosmedicine.org/, Annals of Internal Medicine at http://www.annals.org/, and Epidemiology at http://www.epidem.com/). Information on the STROBE Initiative is available at www.strobe-statement.org.
